# Longitudinal neovascular changes on optical coherence tomography angiography in proliferative diabetic retinopathy treated with panretinal photocoagulation alone versus with intravitreal conbercept plus panretinal photocoagulation: a pilot study

**DOI:** 10.1038/s41433-019-0628-3

**Published:** 2019-11-12

**Authors:** Feng He, Weihong Yu

**Affiliations:** Department of Ophthalmology, Peking Union Medical College Hospital, Key Lab of Ocular Fundus Diseases, Chinese Academy of Medical Sciences and Peking Union Medical College, Beijing, China

**Keywords:** Retinal diseases, Outcomes research

## Abstract

**Purpose:**

To investigate the longitudinal changes in neovascularization of the retinal elsewhere (NVE) size on optical coherence tomography angiography (OCTA) in proliferative diabetic retinopathy (PDR) treated by panretinal photocoagulation (PRP) alone or by single intravitreal conbercept injection plus PRP.

**Methods:**

A prospective pilot study. Forty-four PDR eyes with NVE confirmed by fundus fluorescein angiography (FFA) and OCTA were included. They were assigned to receive PRP alone (PRP group) or intravitreal conbercept injection plus PRP (combination group). Ophthalmic examinations, including BCVA and OCTA to measure the NVE size, were performed at baseline before each PRP session, and at 1, 3, and 6 months.

**Results:**

Twenty-nine eyes were included in the PRP group, and 15 eyes were included in the combination group. There was no significant difference between the two groups with respect to age, BCVA, and NVE area at baseline. In both groups, there was a significant (*P* < 0.05) NVE size reduction during the majority of study visits, with the reduction observed in the combination group significantly larger than that in the PRP group. No significant BCVA changes were observed in either groups, except that in the PRP group, the BCVA at 3 months was significantly improved (*P* < 0.05). No deaths or unexpected adverse events (AEs) were reported.

**Conclusions:**

Intravitreal conbercept plus PRP was more effective than PRP monotherapy in NVE regression. Precise quantification of the NVE area by OCTA makes it a useful tool for monitoring the response of retinal neovascular lesions to the therapy.

## Introduction

Diabetic retinopathy (DR) is the leading cause of visual impairment and blindness in the working population around the world. In China, DR is responsible for vision loss in 12.6% of diabetic patients and tends to occur in patients much younger than those in Western countries [1, 2]. As a severe stage of DR, proliferative diabetic retinopathy (PDR) poses the greatest threat to visual function due to neovascular formation and development [3]. According to the latest Diabetic Retinopathy Clinical Guidelines released by the American Academy of Ophthalmology in 2016 [4], PRP should be performed once the neovascularization (NV) appears, and intravitreal injection of anti-VEGF agents may be recommended until then. It has been confirmed by many authors that PRP combined with anti-VEGF agents, including ranibizumab and bevacizumab, is more effective than PRP alone for NV regression [5–8], but there has been no unified scheme across different studies. In addition, anti-VEGF medications are not commonly covered by medical insurance in China, which makes the repeated injection of such agents unaffordable for many patients. Recently, a single dose of anti-VEGF injection was proven to be effective by several authors; [9, 10] therefore, PRP alone or single-dose anti-VEGF agents combined with PRP could become the practical treatment of choice for our PDR patients with neovascularization.

Conbercept, a new anti-VEGF drug recently developed in China, has a high affinity for all isoforms of VEGF-A, VEGF-B, and placental growth factor. Although conbercept has been used in other diseases with neovascularization [11–13], the use of conbercept in the treatment of PDR is off-label, and there are few reports on this topic. The temporal profile of the response of retinal neovascularization to PRP alone or single-dose conbercept combined with PRP has yet to be established and should be useful for defining the comparative efficacy of different therapy choices as well as the optimal retreatment time.

Fundus fluorescein angiography (FFA) has been the gold standard for evaluating neovascularization of the retinal elsewhere (NVE) over the past decades. However, repetitive FFA acquisitions in PDR patients to observe longitudinal changes in NVE might be impractical due to the invasive and anaphylactic nature of this method. Early and progressive fluorescein leakage from active neovascular tissue may also hinder precise quantification of the NVE area. With the recent introduction of optical coherence tomography angiography (OCTA), the dimensions of NVE can be rapidly quantified without concern for intravenous dye-related adverse outcomes [14], facilitating the regular assessment of PDR patients in their response to different therapies.

Therefore, we conducted this pilot study using OCTA to investigate longitudinal changes in NVE in PDR patients treated with PRP alone or with intravitreal conbercept injection plus PRP. In addition, we sought to compare the short-term and long-term anti-NV efficacy of these two procedures and to identify a promising treatment regimen for PDR patients with economic concerns.

## Methods

This prospective clinical study included patients with clinically diagnosed PDR and active NV who presented at Peking Union Medical College Hospital between May 2017 and May 2018. All patients were informed of the purpose and procedures, and written informed consent was obtained before the treatment. This study was approved by the Ethics Committee of Peking Union Medical College Hospital, and all procedures conformed to the tenets of the Declaration of Helsinki. The inclusion criteria included patients who had PDR and the NVE (which were visible on both FFA and OCTA) and had not received any treatment. Exclusion criteria included fibrovascular proliferation with retinal traction; other causes of NVE, such as retinal vein occlusion; atrophy, scarring, fibrosis, and hard exudates involving the central macula; diabetic macular oedema (DME) with central involvement; and history of vitrectomy, optic neuropathy, and uncontrolled glaucoma.

At baseline, each patient received a comprehensive ophthalmic examination by two experienced retina specialists (FH and WY); the evaluation included best corrected visual acuity (BCVA, Snellen visual acuity ratios) using the International Standard Visual Acuity Chart, intraocular pressure using a TX-20 full auto tonometer (Canon Inc., USA), slit-lamp biomicroscopy, and fundal examination using indirect ophthalmoscopy and OCTA. OCTA images were acquired using the RTVue-XR Avanti system with AngioVue software 2.0 (Optovue Inc., Fremont, California, USA). The “HD Angio Retina 6 × 6 mm” mode was used to encompass the NVE area present on corresponding FFA images, and the NVE size was automatically measured. The OCTA examination and image evaluation were performed by a retina specialist (FH), and low-quality images (signal strength index [SSI] <40) were discarded.

Considering that the use of anti-VEGF agents in PDR patients has not been covered by Chinese public medical insurance, we asked patients to choose the treatment of PRP alone or conbercept plus PRP after a detailed explanation of the procedures and their costs. For the eyes in the PRP group, PRP was performed at one-week intervals according to EDTRS guidelines (Early Treatment Diabetic Retinopathy Study Research Group, 1987) by a single retina specialist (FH) using a VISULAS® Trion multi-wavelength laser (Carl Zeiss Meditec Inc., Dublin, CA). To reduce the occurrence of macular oedema and vitreous haemorrhage, PRP was performed in the order of the nasal, inferior, superior, and temporal sides with 300–400 scatter laser burns per session and was completed after four sessions with a total of 1200–1600 burns. During the treatment, parameters were adjusted to obtain a mild white laser burn with a 300-µm laser and one untreated spot between two burns. For the eyes in the combination group, one intravitreal injection of 0.5 mg/0.05 mL conbercept (Lumit^®^, Chengdu Kanghong Biotech Co., Ltd., P.R. China) followed by 4 PRP sessions was applied. Conbercept was injected intravitreally via a 30-gauge needle inserted through the inferotemporal pars plana 3.5–4.0 mm posterior to the limbus after topical anaesthesia and sterilization, and prophylactic topical antibiotic drops were instilled for 3 days. PRP sessions started one week after the intravitreal injection and were performed exactly the same as that in the PRP group. Patients were examined before each PRP session and at 1, 3, and 6 months after treatment and underwent the same procedures as performed at baseline. The primary outcome measure was NVE regression, which referred to the decrease in NVE area compared with the baseline. The secondary outcome measure was BCVA.

Sample size calculation was not performed for this pilot study, but it was estimated that 30–40 subjects would be sufficient to explore the feasibility of a subsequent study. Statistical analysis was performed using SPSS 20.0 statistical software (SPSS Inc., Chicago, IL). A paired sample *t*-test was used to evaluate the longitudinal changes in BCVA and NVE area after the treatment. An independent sample *t*-test was used to compare the data of two groups at the same time point. A *P* value of <0.05 was considered statistically significant.

## Results

This study enrolled 44 eyes (29 in the PRP group and 15 in the combination group). The patients’ characteristics are summarized in Table 1. There were no statistically significant differences between groups with respect to age, baseline BCVA, and NVE area. No serious adverse events (AEs) were observed in this study.

**Table 1:** Baseline characteristics of patients in two groups

	PRP group	Combination group	*t*	*p*
Eyes (*n*)	29	15	–	–
Mean age (years)	50.8 ± 7.3	50.2 ± 8.3	0.220	0.828
BCVA (Snellen)	0.49 ± 0.69	0.57 ± 0.66	1.074	0.293
NVE area (mm^2^)	1.395 ± 1.139	2.373 ± 2.240	−1.589	0.130

Eyes of the PRP group received seven ophthalmic examinations, that is, before each PRP session and at 1, 3, and 6 months after treatment, whereas eyes in the combination group received one more examination before the intravitreal injection.

**Fig. 1:**
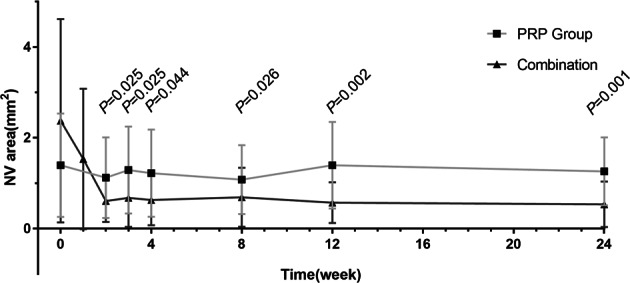
Mean NVE areas (mm^2^) in two groups

A within-group statistically significant reduction in NVE size compared with baseline was found during treatment in the PRP group, and the NVE size at 3 months after treatment was significantly larger (*P* < 0.05). However, there was a statistically significant reduction in NVE size compared with baseline at all time points in the combination group. The inter-group comparison showed a significantly larger reduction in NVE size during and after the treatment in the combination group compared with the PRP group (*P* < 0.05). The mean NVE areas during each examination are summarized in Fig. 1.

**Fig. 2:**
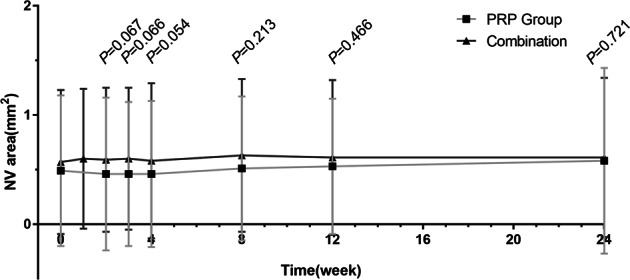
Mean BCVAs (Snellen) in two groups

A significantly improved BCVA compared with baseline was observed at 3 months after treatment in the PRP group (*P* < 0.05), while no statistically significant change in BCVA was observed in the combination group at any time point. Between-group analysis showed no significant difference in BCVA at all time points during and after the treatment (all *P* > 0.05). The mean BCVAs during each examination are summarized in Fig. 2.

No unexpected serious AEs were observed during the study, and no deaths occurred.

## Discussion

In this study, we first describe the temporal profile of the response of NVE to two treatment procedures at the 6-month follow-up. The results of this study suggest that both treatments are associated with significant reduction in NVE areas and improved BCVA to some extent. However, the combination treatment approach proved to be more effective than PRP alone in reducing NVE rapidly and persistently and therefore seems to be an optimal therapeutic regimen for PDR patients, especially for those who cannot afford repeated injections of anti-VEGF drugs.

PRP has been the standard treatment for PDR for more than four decades because it induces NV regression and reduces the risk of severe vision loss [15]. Several authors have used FFA to evaluate the response of NV to PRP at 1–12 months and found that the anti-NV effect of PRP lasted no more than 6 months, although protocols of PRP were different across those studies [16–18]. Our study showed that BCVA at 3 months was significantly improved in these patients. Meanwhile, a significant reduction in NVE appeared as early as 1 week after the first PRP, reached its peak at 1 month, and lasted until 3 months. Such findings added data on the short-term response of NVE to PRP treatment that could not be easily obtained with FFA imaging due to its invasive nature.

**Fig. 3:**
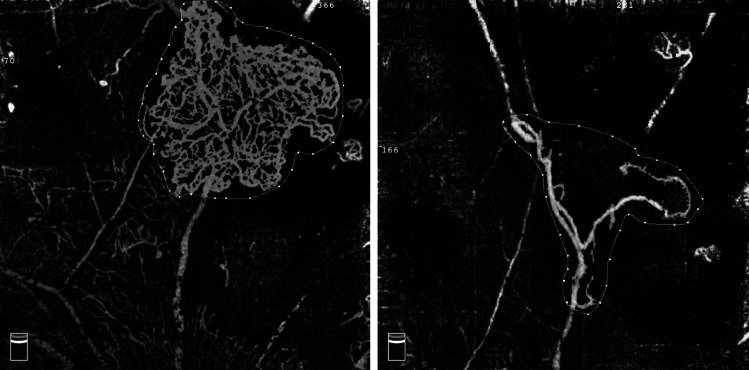
A large group of neovascular vessels is seen at baseline, with an area of 3.976 mm^2^ measured (left); after conbercept intravitreal injection for 1 week, neovascularization is significantly reduced with an area of 0.927 mm^2^ (right)

The use of anti-VEGF drugs has demonstrated a positive effect in regressing NV. It has also been shown that repeated injections of such drugs could improve vision in PDR patients. The main shortcomings of anti-VEGF agents are the short duration and relatively high cost for some patients, but such drugs may also act as adjuvant agents to PRP in improving the response to treatment. A recent multi-centre study by Figueira et al. [7] compared the efficacy of ranibizumab plus PRP vs. PRP alone over a 12-month period and reported a total regression of NV in 43.9% of eyes in the combination group vs. 25.0% in the PRP group, showing the superiority of combination treatment, similarly to other FFA studies [5, 6]. In addition, eyes administered combination treatment might receive fewer laser burns and may be associated with fewer side effects such as pain, macular oedema, and central vision loss. Our study using OCTA to describe NVE changes revealed that the significant reduction in NVE started as early as 1 week after the injection and lasted until 6 months after treatment (Fig. 3). Compared with the PRP group, the NVE reduction observed in the combination group was significantly larger at all study visits, suggesting that combination treatment could cause a quick and durable regression of retinal neovascular tissues. Although only one single dose of anti-VEGF was applied in this combination approach, it showed a synergistic effect with PRP causing an eventual reduction in NVE, as in previous studies using multiple injections [7], suggesting the combination approach to be a cost-effective choice for PDR patients. As for BCVA changes, there was no statistically significant difference between the two groups at any visits, whereas significant visual acuity improvement was only observed in the PRP group at 3 months. Similar findings were reported by Figueira et al. [5, 7]. Possible explanations include the limited number of patients who had fairly good baseline visual acuity but were unassociated with DME. In the Diabetic Retinopathy Clinical Research network study [19], 35% of cases in the PRP group were given ranibizumab at baseline due to DME, and this treatment might be related to the significantly improved BCVA reported in this study.

Conbercept, which is an anti-VEGF drug developed in China in recent years, was used in this study for intravitreal injection. It has been approved by the China Food and Drug Regulatory Administration for the treatment of neovascular age-related macular degeneration and was recently admitted directly into Phase III clinical trials by the U.S. Food and Drug Administration. Conbercept competitively prevents the binding of VEGF to its receptor, inhibits downstream pathway activation, and has a higher binding affinity to the most potent pro-angiogenic alternative splicing form of VEGF-VEGF-A than other widely used anti-VEGF drugs [20]. There are relatively few reports on conbercept use for reducing NV in PDR patients except that it has been used for preoperative injection and for other diseases involving neovascularization [11–13]. We found that the intravitreal injection of conbercept combined with PRP could achieve a significant reduction in NVE areas within 6 months, and this approach proved to be an effective and safe treatment for PDR patients. In addition, as conbercept has a longer intravitreal clearance time than other anti-VEGF agents, its long-term benefits over other agents still need to be elucidated.

**Fig. 4:**
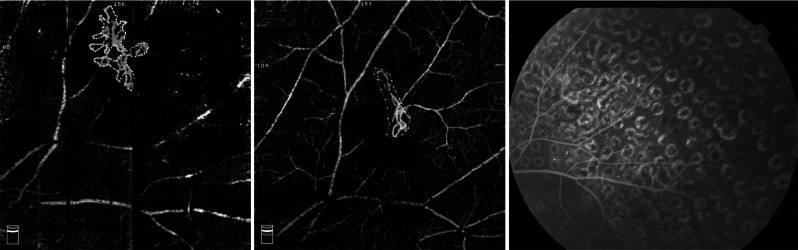
Neovascularization at baseline OCTA with an area of 0.653 mm^2^ (left); OCTA still showed abnormal vascular shape at an interval of 1 year after PRP, with an area of 0.272 mm^2^ (middle), and no evidence of fluorescein leakage was seen at the FFA (right)

To the best of our knowledge, this is also the first study to use OCTA to observe longitudinal changes in NVE treated by different methods. Previous studies have described preretinal NV in PDR using OCTA [21] and have reported the temporal profile of the response of neovascularization of the disc size to a single intravitreal bevacizumab injection within a 30-day period [9]. The changes in NVE over time could be established with frequent and repeated OCTA examination, which is impossible when using FFA due to its invasive nature. The current limitations of OCTA imaging lie in the following aspects. First, accurate NVE assessment could hardly be achieved when the NVE size is beyond the detectable range of the instrument. Second, the absence of signals on OCTA indicates the absence of flow but does not exclude patent vessels with very slow blood flow below the detection threshold, thus leading to the underestimation of NVE. Third, in some cases, there might be disagreements between OCTA and FFA in the diagnosis of NVE. In one of our cases, shrinking NVE was present on OCTA images 1 year after PRP, but no evident fluorescein leakage was observed on FFA (Fig. 4), suggesting that the function of endothelial cells in these residual blood vessels may have improved. Since not all included cases were followed by FFA beyond 6 months after treatment, whether this phenomenon generally exists or not as well as how these blood vessels may develop remains unclear.

There are some limitations to this pilot study. First, the sample size and short follow-up time of 6 months did not allow a comprehensive evaluation of the changes in NVE due to different treatments. Second, eligible participants were not randomly divided into two groups because of the economic concerns of some patients; under this circumstance, the treatment choice was chiefly based on their wishes. Further randomized studies would reinforce the results obtained in this study.

In summary, we observed in this study that the combination of intravitreal conbercept injection with PRP resulted in a higher reduction rate of NVE than PRP alone in PDR patients. OCTA has an important role in visualizing the NVE area and monitoring its response to therapies. Finally, larger studies with longer follow-up are required to ascertain our preliminary findings.

## Summary

### What was known before

- PRP combined with repeated anti-VEGF agents was more effective than PRP alone for NV regression in PDR.
- The repeated injection of anti-VEGF agents is unaffordable in many patients in China.

### What this study adds

- This is the first study that used OCTA to observe the longitudinal changes of NVE treated by different methods in PDR.
- This study firstly describe the temporal profile of the response of NVE to two treatment procedures at 6 months follow-up.
- Combination of single-dose intravitreal Conbercept injection with PRP resulted in a higher reduction rate of NVE than PRP alone in PDR.
